# Supplementary material for: SARS-CoV-2 positivity rates associated with circulating 25-hydroxyvitamin D levels
Source: PLoS One. 2020 Sep 17;15(9):e0239252. doi: 10.1371/journal.pone.0239252 (PMC7498100; doi:10.1371/journal.pone.0239252)
Supplement: Attachment — Submitted filename: Response to Editors 8.26.20.docx [file pone.0239252.s001.docx]

Dear Dr. Holick,

Thank you for submitting your manuscript entitled "SARS-CoV-2 Positivity Rates Associated with Circulating 25-Hydroxyvitamin D Levels" to PLOS ONE.  Your manuscript files have been checked in-house but before we can proceed we need you to address the following issues:

(1) Thank you for providing an updated Funding statement. We've made a minor change to meet our requirements, can you please confirm whether the following proposed statement is accurate and suitable to appear alongside your manuscript?

"Quest Diagnostics provided support in the form of salaries for authors JKN, BC, MHK, and HWK and consulting fees for MFH but did not have any additional role in the study design, data collection and analysis, decision to publish, or preparation of the manuscript. The specific roles of these authors are articulated in the ‘author contributions’ section.”

We will update the statement on your behalf with your approval.

**Author’s response:** Thank you, we approve, please provide middle initial (MHK) for author as well.


(2) We're also requesting the following update to your Competing Interest statement:

"HWK, JKN, MHK, and CB are employees of Quest Diagnostics. HWK, MHK and CB own stock in Quest Diagnostics. MFH is a consultant to Quest Diagnostics and was on the speakers’ bureau for Abbott Inc. and Hyatt Pharmaceutical Industries Company PLC. There are no patents, products in development or marketed products associated with this research to declare. This does not alter our adherence to PLOS ONE policies on sharing data and materials."

If any of this information is incorrect, please clarify, otherwise we will update the statement with your approval.

**Author’s Response:** Please see the response below for our statement on data sharing. If this conforms to your policies then the updated competing interest statement is approved.


(3) We note that you've stated the following regarding your data: "The data underlying the results presented in the study are available from Quest Diagnostics Clinical Laboratory Database and are stored in the Quest Diagnostics Informatics Data Warehouse.”

PLOS only allows data to be available upon request if there are legal or ethical restrictions on sharing data publicly. For information on unacceptable data access restrictions, please see <https://nam04.safelinks.protection.outlook.com/?url=http%3A%2F%2Fjournals.plos.org%2Fplosone%2Fs%2Fdata-availability%23loc-unacceptable-data-access-restrictions&amp;data=02%7C01%7CHarvey.W.Kaufman%40questdiagnostics.com%7C204b422f20524f74c4bd08d849bc2198%7Cb68c6481b22b46b38c4c0024bb9b9b1f%7C1%7C0%7C637340419657309411&amp;sdata=7dkabK3hbEyGDtegn0XYB5rParh4lkIFSbeny812%2Bsc%3D&amp;reserved=0>.

Before we proceed with your manuscript, please address the following prompts:

a) If there are ethical or legal restrictions on sharing a de-identified data set, please explain them in detail (e.g., data contain potentially identifying or sensitive patient information, data are owned by a third-party organization, etc.) and who has imposed them (e.g., a Research Ethics Committee or Institutional Review Board, etc.). Please also provide contact information for a data access committee, ethics committee, or other institutional body to which data requests may be sent.

b) If there are no restrictions, please upload the minimal anonymized data set necessary to replicate your study findings to a stable, public repository and provide us with the relevant URLs, DOIs, or accession numbers. Please see <https://nam04.safelinks.protection.outlook.com/?url=http%3A%2F%2Fwww.bmj.com%2Fcontent%2F340%2Fbmj.c181.long&amp;data=02%7C01%7CHarvey.W.Kaufman%40questdiagnostics.com%7C204b422f20524f74c4bd08d849bc2198%7Cb68c6481b22b46b38c4c0024bb9b9b1f%7C1%7C0%7C637340419657309411&amp;sdata=1tq7CqcqrkL%2F6Ke9QspE%2FQzIGfCmtiNp9rs1tYyFN9I%3D&amp;reserved=0> for guidelines on how to de-identify and prepare clinical data for publication. For a list of recommended repositories, please see <https://nam04.safelinks.protection.outlook.com/?url=https%3A%2F%2Fjournals.plos.org%2Fplosone%2Fs%2Frecommended-repositories&amp;data=02%7C01%7CHarvey.W.Kaufman%40questdiagnostics.com%7C204b422f20524f74c4bd08d849bc2198%7Cb68c6481b22b46b38c4c0024bb9b9b1f%7C1%7C0%7C637340419657309411&amp;sdata=Og4xLiA%2Fo0ERih%2Byo5fBPwpPWaRkTX3XmJRFeu%2BwJFU%3D&amp;reserved=0>. You also have the option of uploading the data as Supporting Information files, but we would recommend depositing data directly to a data repository if possible.

We will update your Data Availability statement on your behalf to reflect the information you provide.

**Authors’ Response:** In accordance with Quest Diagnostics company policy on data security we are not able to upload the 191,000 rows of data to a public repository. We cannot violate HIPAA and open our Data Informatics Warehouse to external parties. This legal restriction applies to academic institutions as well as to our commercial organization.

Quest Diagnostics has previously published in PLoSONE:

<https://journals.plos.org/plosone/article?id=10.1371/journal.pone.0180840>

<https://journals.plos.org/plosone/article?id=10.1371/journal.pone.0118108>

<https://journals.plos.org/plosone/article?id=10.1371/journal.pone.0063416>

<https://journals.plos.org/plosone/article?id=10.1371/journal.pone.0028201>

In all prior circumstances as with this study, we have made data available to researchers upon request. We are eager to have others validate our analysis, suggest new ways to analyze and interpret our data, and explore how we can further understanding of health and disease. In recent months we have worked on other subjects with investigators from many outside organizations including the CDC, Boston University, Penn State University, the University of Alabama, the Alameda Health System, and many others. Requests for data and research collaborations can be sent to HealthTrends@QuestDiagnostics.com

(4) In the Methods section of your manuscript, please include the following statement: "HIPAA clearly defines research use of data as analyzed for this and numerous other studies based on the Quest Diagnostics Data Informatics Warehouse (45 CFR 164.501, 164.508, 164.512(i) (See also 45 CFR 164.514(e), 164.528, 164.532) Link: <https://nam04.safelinks.protection.outlook.com/?url=https%3A%2F%2Fwww.hhs.gov%2Fhipaa%2Ffor-professionals%2Fspecial-topics%2Fresearch%2Findex.html&amp;data=02%7C01%7CHarvey.W.Kaufman%40questdiagnostics.com%7C204b422f20524f74c4bd08d849bc2198%7Cb68c6481b22b46b38c4c0024bb9b9b1f%7C1%7C0%7C637340419657314405&amp;sdata=R9Yjj38kKasss73QqHM0u2z16rLwk64VX3QAImAFrRg%3D&amp;reserved=0> ). Quest Diagnostics takes the additional step of having its process reviewed annual by the Western Institutional Review Board who has determined the process is “deemed exempt."

**Authors’ Response:** We have added this statement to the methods section.

Your manuscript has been returned to your account. Please log on to PLOS Editorial Manager at <https://nam04.safelinks.protection.outlook.com/?url=https%3A%2F%2Fwww.editorialmanager.com%2Fpone%2F&amp;data=02%7C01%7CHarvey.W.Kaufman%40questdiagnostics.com%7C204b422f20524f74c4bd08d849bc2198%7Cb68c6481b22b46b38c4c0024bb9b9b1f%7C1%7C0%7C637340419657314405&amp;sdata=%2F2Lb3ZZU82hslYEkPeYh8KeqjCgaz5Yz8r9Olq47s%2BU%3D&amp;reserved=0> to access your manuscript.

Your manuscript can be found in the "Revisions Sent Back to the Author" link under the New Submissions menu. After you have made the changes requested above, please be sure to view and approve the revised PDF after rebuilding the PDF to complete the resubmission process.

Please note that these changes have been requested to comply with submission guidelines and your manuscript will *not* be sent to review until you have fully adhered to our requests.  Once your paper has been seen by an Editor we may return it to you for further information or amendments.

We ask that you address this request within 28 days. If you require additional time, please email the journal office. We are happy to grant extensions of up to one month past this due date. If we have not heard from you within 28 days, your manuscript will be withdrawn from Editorial Manager.

Thank you for submitting your work to PLOS ONE.

Kind regards,

Agnes Magyar
PLOS ONE
